# Pulmonary function, body posture and balance in young adults with asthma: A cross-sectional study

**DOI:** 10.1371/journal.pone.0316663

**Published:** 2025-03-03

**Authors:** Gopala Krishna Alaparthi, Ibrahim Moustafa Moustafa, Agnaldo José Lopes, Arthur de Sá Ferreira

**Affiliations:** 1 Augusto Motta University Center, UNISUAM, Rio de Janeiro, Brazil; 2 Department of Health Professions, Manchester Metropolitan University, Manchester, United Kingdom; 3 Department of Physiotherapy, College of Health Sciences, University of Sharjah, M23, Medical College, Sharjah, United Arab Emirates; 4 School of Medical Sciences, State University of Rio de Janeiro (UERJ), Rio de Janeiro, Brazil; National Trauma Research Institute, AUSTRALIA

## Abstract

Asthma may lead to musculoskeletal dysfunction, including postural abnormalities. This study aimed to compare pulmonary function, body posture, and dynamic balance in young adults with childhood-onset asthma and healthy peers. This cross-sectional study included 124 participants evenly split between asthma and healthy groups. Assessments covered pulmonary function, respiratory muscle strength, postural balance, and body posture. We observed differences between groups in pulmonary function variables and respiratory muscle strength (Forced Expiratory Volume in the First Second (FEV 1); p = 0.005, forced vital capacity (FVC);.p = 0.002, peak expiratory flow rate (PEFR); p = 0.03) and respiratory muscle strength (maximal. expiratory pressure (MEP); p = 0.05). There was no difference in postural balance and body posture between groups. Moderate correlations were noted between postural balance (double leg eye closed) and pulmonary function (r = 0.38-0.43; p < 0.001), but not between body posture and pulmonary function. These findings suggest childhood-onset asthma in young adults, particularly mild to moderate cases, is associated with impaired pulmonary function and respiratory muscle strength but does not significantly impact postural balance or body posture compared to healthy peers.

**Trial registration:** ClinicalTrials.gov NCT05373511

## 1. Introduction

Asthma, a prevalent chronic respiratory condition characterized by persistent airway inflammation, predominantly affects children [1,2]. While it can develop at any age, onset typically occurs during childhood, presenting with varying severity [3]. During asthma attacks, hypersensitivity leads to increased airway resistance, air trapping, and elevated end-expiratory lung volume. Consequently, the diaphragm undergoes deformation, restricting rib cage expansion and impairing inspiratory muscle function, particularly that of the diaphragm [4]. The diaphragm also contributes to trunk stabilization, a crucial aspect of postural stability [5]. Asthma is linked to musculoskeletal dysfunction and postural abnormalities [6,7]. Individuals with persistent asthma often exhibit a postural pattern characterized by head protraction and elevation, shoulder girdle protraction, reduced chest wall expansion, and shortening of upper arm and posterior trunk muscles [8]. Physical inactivity associated with asthma tends to impact posture, resulting in increased thoracic kyphotic depth among inactive asthmatic individuals [8]. Postural deviations correlate with airway obstruction variables like hyperventilation and air trapping [9].

These postural changes may contribute to impairments in postural balance among individuals with asthma [5,10–12]. Variations in muscle activation sequence, delayed recruitment of synergistic muscles, activation of antagonist muscles, delayed initiation of postural responses, and variations in muscle response intensity can all influence balance [13]. These alterations can lead to a diminished ability to perceive and regulate mid-lateral and anterior-posterior body movements. Evidence suggests that patients with lung disorders, including asthma, often exhibit significant deficits in postural balance, with underlying multifactorial pathophysiological mechanisms [9,12,14].

Surface topography, which involves analyzing the three-dimensional surface area of the back, has gained popularity for assessing postural abnormalities [15]. Similarly, the Biodex balance system offers a cost-effective and portable option with adjustable platforms to provide different levels of stability for the assessment and treatment of balance conditions [16–18]. Studies have indicated postural and balance abnormalities among young adults, albeit with methodological limitations such as diverse assessment methods and lack of high-reliability outcomes [12,14,16,19]. A study exploring the relationship between pulmonary function and postural balance revealed a limited understanding of this subject [10]. Therefore, there is a pressing need for high-quality research employing valid and reliable clinical outcome measures for both body posture and postural balance. Our study provides objective measurements of both balance and posture in young adults with childhood-onset asthma, which were previously lacking in the literature.

The primary objective of the study is to describe body posture changes in young adults with childhood-onset asthma and compare them to a non-asthmatic control matched group and to describe the dynamic balance of young adults with childhood-onset asthma and compare them to a non-asthmatic control matched group. The secondary objective is to examine the relationship between pulmonary function, body posture, and dynamic balance in young adults with childhood-onset asthma. We Hypothesis that young adults with childhood-onset asthma will demonstrate lower pulmonary function and reduced respiratory muscle strength compared to healthy controls. Additionally, it is hypothesized that there will be significant differences in postural balance and body posture between the two groups, but correlations will exist between pulmonary function postural balance and body posture in the asthma group.

## 2. Materials and methods

### 2.1 Ethics

The University of Sharjah’s Institutional Ethics Committee approved the study (approval number REC-22-01-12, approval date March 16, 2022). Before enrolling patients in the trial, the aim of the study was explained to them and written informed consent was obtained.

### 2.2 Study design

Correlational cross-sectional study. This study was prospectively registered under ClinicalTrials.gov (NCT05373511).

### 2.3 Setting and participants

The study was conducted at the College of Health Sciences, University of Sharjah, United Arab Emirates, from 15th April 2022 to 28th April 2023.

Inclusion criteria for the asthma group comprised individuals aged 18 to 25 years with mild to moderate asthma since childhood, referred by a pulmonologist or physician, medically stable, and without exacerbations within the previous 6 weeks. The control group included volunteers referred by a consultant pulmonologist/physician, matched for age, gender, weight, and height. Exclusion criteria for both groups included other health conditions such as cardiorespiratory issues, neurological deficits, or diseases; history of severe musculoskeletal injuries; developmental disorders (e.g., juvenile osteochondrosis, adolescent idiopathic scoliosis); notable postural abnormalities; spine-related infections, tumors, or surgeries; and vestibular disorders.

### 2.4 Assessments

All subjects underwent body composition assessment using an electrical bioimpedance method, while pulmonary function and respiratory muscle strength tests followed guidelines [20–22]. Participants received an explanation of the functioning of the postural analysis and postural balancing machines. Subsequently, participants underwent a postural scan for body posture assessment [23–25]. Following the body posture assessment, participants remained fully dressed for the postural balancing test utilizing the Biodex balance system machine. [16,17]. The total duration of the assessments was approximately 50-60 minutes per participant, allowing sufficient time for each test while preventing participant fatigue.

### 2.5 Body composition

Body composition was assessed using an electrical bioimpedance method with a calibrated segment analyzer (Tanita MC-980 PLUS MA, Tokyo, Japan). The analyzer features eight electrodes, with four integrated into the platform and the remainder in holders. Participants assumed a motionless position on the platform within designated zones (26). Participants stood barefoot with straight legs on the platform, ensuring their feet contacted both front and back electrodes, and distributed their body weight evenly between both feet. Holding the handles at an angle of 35°-40° away from the body, participants’ height was measured while standing, with electrodes in contact with their bare feet and hands. The device computed body fat percentage using equation formulas based on measured body weight, impedance, the participant’s sex, body height, fitness, age, and weight [26]

### 2.6 Pulmonary function test

Pulmonary function tests were conducted using the MIR-Spiro Lab Spirometer (Medical International Research, Italy, 2020). The recorded variables included the best value of three acceptable tests for forced vital capacity (FVC), Forced Expiratory Volume in the First Second (FEV1), and peak expiratory flow rate (PEFR) [20,27].The study reported consistently good Intraclass Correlation Coefficients (ICC) >  0.75 for all measured variables[22].

### 2.7 Respiratory muscle strength test

Maximal inspiratory pressure (MIP) and maximal expiratory pressure (MEP) were measured using a MicroRPM device (CareFusion UK 232 Ltd, Basingstoke, UK). Subjects remained seated with their trunk at a 90° angle to their hips and their feet on the ground. During all maneuvers, subjects wore a nasal clip to prevent air leakage. Subjects were instructed to perform a maximal inspiratory effort starting from residual volume (RV) for MIP and a maximal expiratory effort starting from total lung capacity (TLC) for MEP. Each subject completed at least three reproducible maneuvers, each lasting at least one second, until three technically competent efforts were achieved. A one-minute rest period was provided between efforts. If the highest value did not exceed the second highest value by 10%, it was considered for data analysis [21,22,28]

### 2.8 Outcomes: postural balance

The Biodex Balance System (BBS) (Manufactured by Biodex Medical Systems, Inc. in the United States) was utilized to evaluate balance and postural stability under dynamic stress. They stood on a platform and were instructed to maintain balance so that the cursor on the displayed screen/monitor remained at the center of a co-centered circle with the ability to tilt up to 20°, the BBS calculates three key measures: Medial-Lateral Stability Index (MLSI), Anterior-Posterior Stability Index (APSI), and Overall Stability Index (OSI). A higher OSI score indicates poorer balance, serving as a strong predictor of overall balancing ability. Adjusting the resistance level of the springs beneath the platform modifies its stability, with a scale ranging from 1 to 8, where 1 reflects maximal instability.Participants performed bilateral stance tasks with both eyes open and closed for 20 seconds. Stability levels were adjusted from level 6 to level 3, and participants were instructed to keep their center of pressure within the smallest concentric rings (balancing zone) displayed on the BBS monitor. Initially, participants stood on the locked platform, and the stability platform was then unlocked to allow movement, enabling the determination of the optimal foot position for testing. Once found, the foot position was fixed for the duration of the session.

The platform was released for a 20-second trial, during which participants were required to maintain an erect standing stance on their limbs. Successful completion of the trial necessitated maintaining balance for the entire duration. To familiarize participants with the equipment and mitigate learning effects, all participants underwent 1 minute of training followed by three practice trials. Three test evaluations were then conducted, with scores averaged across the trials. The Romberg overall score was calculated [16,17,29,30].

### 2.9 Outcomes: body posture

**Fig 1:**
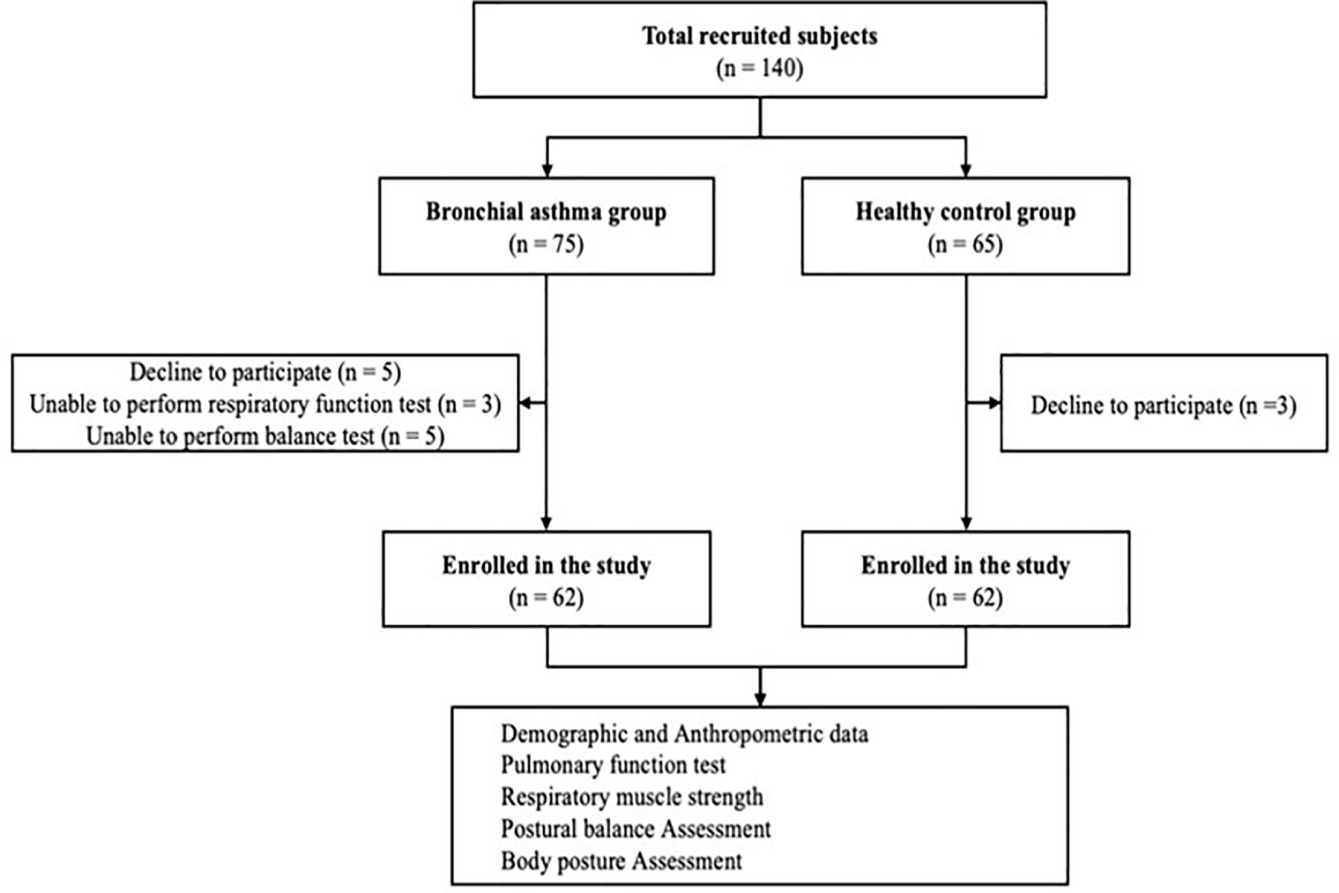
Consort flow diagram of the study.

DIERS Formetric 4D® (Manufactured by DIERS International GmbH in Germany) sensors were employed to detect surface topography across the entire back while participants maintained a static standing posture (Fig 1). To ensure privacy and maintain a quiet environment, the laboratory remained silent throughout the procedure. Subjects were instructed to remove their T-shirts and stand in their usual relaxed standing position facing away from the machine’s window. Once properly positioned, the equipment captured photographs that were converted into 3D images on a computer. Reflective stickers were carefully affixed to three bony landmarks—specifically, the C7 spinous process and the right and left posterior superior iliac spines (PSIS), commonly known as sacral dimples—by a physiotherapist proficient in spinal palpation and postural assessment. Participants were guided to locate the C7 spinous process by minimally flexing their necks.

**Fig 2:**
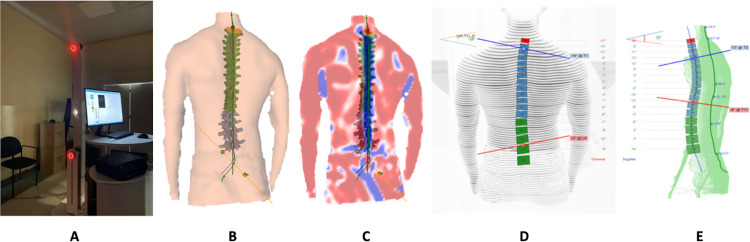
A. The DIERS formetric 4D® device for assessment of body posture. B-E: calculation of body posture lengths, alignments, angles, and rotations.

Participants were instructed to stand barefoot with their heels aligned with the treadmill’s reference line, approximately 220 cm from the DIERS Formetric 4D® device’s light projector (see Fig 2). Facing the cameras, participants laid their backs on them, maintaining a static head posture with their eyes fixed on a reference point adjusted according to their height. Participants’ bodies were exposed from the occiput to the beginning of the inter-gluteal cleft. Using the DiCAM III software, the spinal position was scanned three times, with each scan lasting approximately 6 seconds at a frequency of 2 Hz. During each scan, 12 images were captured, totaling 40 spine form parameters for evaluation. These parameters, automatically obtained and digitized by the equipment, were classified into five categories based on clinical significance: localization and distance, trunk and pelvic imbalances, spinal reference points, spinal curve measures, and spinal deviation (23–25). Supplementary File 2 shows the postural analysis parameters and their definitions.

### 2.10 Sample size & statistical analysis

We used R project version 4.3.0 to conduct a sample size calculation. The calculation was based on a pilot study involving 5 participants from each group, totaling 10 samples. Considering a type-I error of 5% and aiming for a study power of 80%, we computed the required sample size to observe effect sizes (Cohen’s d) equal to or as extreme as those identified in the pilot study for posture and postural balance variables. To maximize the expected coverage, the largest sample size was chosen. A total of 124 participants, with 62 individuals per group, was determined as the required sample size. Prior to conducting the main analyses, we assessed the distribution of all variables using the Shapiro-Wilk test. Descriptive summaries were presented as mean (SD) or absolute frequency (%). Between-group comparisons were executed utilizing the independent samples t-test or the Mann-Whitney test as appropriate. Cohen’s d was computed as an effect size measure for these comparisons. Pearson correlation coefficients, accompanied by 95% confidence intervals, were computed to investigate the correlation of dynamic balance and body posture variables with respiratory function. Statistical significance was defined as p < 0.05.

## 3. Results

Initially, 140 individuals were recruited, with 75 in the asthma group and 65 in the healthy control group. Thirteen asthmatic participants and three healthy subjects were subsequently excluded for various reasons. Following completion of all assessments, the study included 124 participants, with 62 patients with asthma compared to 62 healthy matched controls.

The mean age was 21.4 (2.25) for the asthma group and 21.5 (2.09) for the healthy control group. The majority of subjects in the asthma group (82.3%) were females. Table 1 presents a comparison of body composition between the two groups. Only fat mass (%) and fat mass (kg) exhibited a statistically significant difference (p < 0.05) between the groups (Table 1).

**Table 1:** Demographic characteristics and comparison of body composition between asthma and control groups.

Variable: mean (SD)	Asthma group (n = 62)	Control group (n = 62)	Overall (n = 124)	Effect size	p-value
**Gender**					
Male	11 (17.7%)	33 (53.2%)	44 (35.5%)	0.354	**<0.001**
Female	51 (82.3%)	29 (46.8%)	80 (64.5%)		
**Age (years)**	21.4 (2.25)	21.5(1.92)	21.5 (2.09)	-0.039	0.977
**Height (m)**	1.66 (0.09)	1.69 (0.09)	1.67 (0.09)	-0.332	0.187
**Weight (kg)**	68.5 (18.1)	68.4 (17.0)	68.4 (17.5)	0.00	1.000
**Body mass index (kg/m**^2^)	24.7 (5.74)	23.7 (4.34)	24.2 (5.10)	0.205	0.521
**Muscle mass (kg)**	45.7 (12.0)	49.6 (12.6)	47.6 (12.4)	-0.317	0.215
**Bone mass (kg)**	2.56 (0.61)	2.59 (0.60)	2.58 (0.61)	-0.055	0.954
**Fat-free mass (kg)**	48.6 (12.2)	51.1 (12.9)	49.8 (12.6)	-0.201	0.537
**Fat mass (kg)**	22.3 (11.4)	17.5 (7.93)	19.9 (10.0)	0.490	0.028
**Fat mass (%)**	30.3 (9.90)	24.8 (8.17)	27.6 (9.46)	0.606	0.005
**Smoker**					
No	47 (75.8%)	45 (72.6%)	92 (74.2%)	0.08	0.919
Yes	15 (24.2%)	17 (27.4%)	32 (25.8%)		
**Duration of asthma**					
>15 years	39 (63%)	NA	–	–	–
<15 years	23 (37%)				
**Mild**	33 (53%)	NA	–		–
**Moderate**	29 (46.7%)			–	

**Table 2:** Comparison of pulmonary function variables and respiratory muscle strength between asthma and control groups.

Variable mean (SD)	Asthma group (n = 62)	Control group (n = 62)	Overall (n = 124)	Effect size	p-value
**FVC (L)**	3.06 (0.89)	3.71 (1.05)	3.39 (1.02)	−0.667	**0.002**
**FVC (% predicted)**	80.5 (20.9)	87.5 (17.2)	84.0 (19.4)	−0.365	0.132
**FEV**_1_ **(L)**	2.24 (0.83)	2.80 (1.02)	2.52 (0.97)	−0.606	0.005
**FEV**_1_ **(% predicted)**	67.1 (24.7)	75.7 (21.9)	71.4 (23.6)	−0.368	0.128
**FEV**_1_ **/FVC (%)**	72.6 (17.7)	76.7 (16.6)	74.6(17.2)	−0.238	0.417
**FEV**_1_ **/FVC (% predicted)**	82.8 (21.4)	86.6 (18.6)	84.7 (20.1)	−0.189	0.575
**PEFR (L/min)**	3.38 (1.73)	4.29(2.03)	3.84 (1.93)	−0.484	0.031
**MIP (cmH**_2_ **O)**	59.9 (17.1)	60.3 (19.6)	60.1 (18.3)	−0.021	0.993
**MEP (cmH**_2_ **O)**	62.8 (14.2)	69.8 (17.8)	66.3 (16.4)	−0.436	0.058

FVC: Forced vital capacity, FEV_1_: Forced expiratory volume in the first second, PEFR: Peak expiratory flow rate, MIP: Maximal inspiratory pressure, MEP: Maximal expiratory pressure.

Table 2 compares pulmonary function variables and respiratory muscle strength between asthma and the control groups. The results revealed a statistically significant difference between the groups except for maximal inspiratory pressure (p > 0.05).

Table 3 compares the postural balance variables between asthma and control groups. The results showed that there was a slight discrepancy between the mean values of asthma and control groups, the differences were not statistically significant (p > 0.05).

**Table 3:** Comparison of postural balance between asthma and control groups.

Variable: mean (SD)	Asthma group (n = 62)	Control group (n = 62)	Overall (n = 124)	Effect size	p-value
**Double leg support, eyes open**					
Overall stability index (degree)	3.10 (2.12)	2.87 (1.55)	2.99 (1.86)	0.121	0.798
Anterior-posterior stability index (degree)	2.57 (1.94)	2.40 (1.39)	2.48 (1.68)	0.102	0.850
Medial-lateral stability index (degree)	1.73 (0.95)	1.56 (0.87)	1.65 (0.91)	0.194	0.560
**Double leg support, eyes closed**					
Overall stability index (degree)	5.79 (2.75)	5.71 (3.07)	5.75 (2.90)	0.028	0.988
Anterior-posterior stability index (degree)	4.81 (2.41)	4.68(2.64)	4.74 (2.52)	0.054	0.956
Medial-lateral stability index (degree)	3.20 (1.82)	3.20 (1.73)	3.20 (1.77)	−0.003	1.000

Table 4 compares body posture variables between asthma and control groups. The results showed that there was a slight discrepancy between the mean values of asthma and the control group, however, the differences were not statistically significant (p > 0.05) except in trunk length and kyphotic angle (p < 0.05).

**Table 4:** Comparison of body posture between asthma and control groups.

Variable: mean (SD)	Asthma group (n = 62)	Control group (n = 62)	Overall (n = 124)	Effect size	p-value
**Localization and distance**					
Trunk length (mm)	428 (46.7)	451 (53.1)	440 (51.1)	−0.458	**0.043**
Dimple distance (mm)	87.2 (21.4)	86.8 (18.0)	87.0 (19.7)	0.022	0.992
**Trunk and pelvic imbalances**					
Sagittal imbalance, Anterior (degree)	2.68 (2.63)	1.85 (2.32)	2.27 (2.50)	0.332	0.186
Sagittal imbalance, posterior (degree)	1.11 (2.06)	1.05 (1.96)	1.08 (2.00)	0.032	0.984
Coronal imbalance, right (mm)	6.02 (8.07)	5.65 (7.96)	5.83 (7.99)	0.046	0.967
Coronal imbalance, Left (mm)	5.16 (9.28)	4.13 (6.41)	4.65 (7.96)	0.129	0.771
Pelvic obliquity, right (mm)	1.15 (2.54)	1.47 (2.39)	1.31 (2.46)	−0.131	0.767
Pelvic obliquity, left (mm)	2.77 (3.01)	1.63 (2.48)	2.20 (2.81)	0.415	0.074
Pelvic torsion, right (degree)	1.24 (1.80)	1.53(1.94)	1.39 (1.87)	−0.15	0.688
Pelvic torsion, left (degree)	1.06 (1.66)	1.24(2.04)	1.15 (1.85)	−0.095	0.868
**Spinal curve measurements**					
Kyphotic angle (max) degree	51.7 (10.3)	47.0 (8.83)	49.3(9.85)	0.499	**0.025**
Lordotic angle (max) degree	49.3 (12.4)	44.9 (12.4)	47.1 (12.3)	0.356	0.146
**Spinal deviations**					
Vertebral rotation (rms) degree	3.56 (2.43)	3.55 (2.29)	3.56(2.35)	0.007	0.999
Vertebral rotation (+max) degree	4.94 (5.40)	5.68 (4.25)	5.31 (4.85)	−0.153	0.697
Vertebral rotation (-max) degree	4.03 (3.89)	3.18 (3.37)	3.60 (3.65)	0.235	**0.427**
Vertebral rotation (amplitude) degree	8.98 (5.23)	8.85 (4.33)	8.92 (4.78)	0.027	0.989

**Fig 3:**
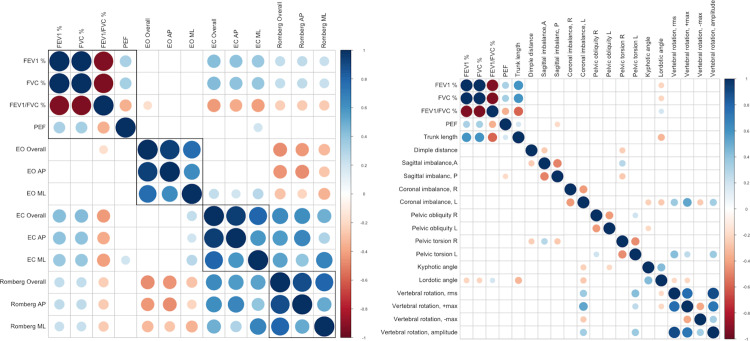
Correlation plot analysis between pulmonary function, body posture, and postural balance variables.

Fig 3 and Supplementary Files 1 and 2 describe the correlation between pulmonary function and postural balance, as well as the relationship between pulmonary function and body position. A significant moderate correlation was found between pulmonary function variables (predicted values of FVC, FEV_1_, FEV_1_/FVC, and PEFR) and postural balance (overall stability index, anterior-posterior stability index, medial-lateral stability index) double leg support, eye closed. Body posture assessment parameters also exhibited significant weak-to-moderate correlations with pulmonary function variables (Fig 3).

## 4. Discussion

The study’s findings comprise differences in pulmonary function and respiratory muscle strength between groups, although postural balance exhibited a minimal effect size without statistical significance; aside from trunk length and kyphotic angle, no significant variations were observed in body posture metrics across groups; and a weak-to-moderate relationship was identified between postural balance variables during double leg eye closure and pulmonary function parameters. Conversely, there was no significant correlation between most body posture variables and pulmonary function, except for a few, such as trunk length and lordotic angle, which displayed a weak to moderate relationship. These findings contradict clinical evidence suggesting alterations in postural balance and body posture among young adults with mild to moderate asthma.

Differences in respiratory function were observed across groups for all pulmonary function variables (FEV1, FVC, PEFR) and respiratory muscle strength (MEP). Hyperinflation in asthma may lead to the flattening of the diaphragm, resulting in mechanical disadvantages and reduced function of the inspiratory muscles. The impaired expiratory muscle strength may contribute to the airflow limitation characteristic of asthma, as these muscles play a crucial role in forced expiration and cough effectiveness. This finding aligns with previous research, which indicates that in asthma patients, hyperinflation causes the shortening of the inspiratory muscles, thereby limiting their efficacy [9,10,14].

The asthma group exhibited lower postural balance compared to the healthy control group, particularly evident during the double leg support, and eye closed condition, as indicated by lower scores in the overall stability index, anterior-posterior stability index, and medial-lateral stability index. This impairment in postural balance among asthma patients may be attributed to the degree of hyperinflation, which could lead to increased activation of trunk muscles and subsequent rigidity. This heightened muscular activation may diminish the significance of trunk motion and force moments in balance control [9–12].

Another potential explanation for decreased postural balance in asthma patients is their heightened reliance on visual input for balance control on a mobile platform. Due to potential deficits in motor systems, they may use visual information more heavily to compensate for instability, which could make balance more challenging in environments where visual input is limited or altered [30]. Previous studies have reported an increased area of center of pressure displacement in asthma patients when exposed to a force platform. Stability is achieved through the generation of force moments on the body joints, countering the effects of gravity and other perturbations. This dynamic process sustains a specific posture over time, necessitating the integration of somatosensory and vestibular information for effective postural control. Ankle torque contributes to anteroposterior balance maintenance, while hip and trunk torque plays a role in mediolateral equilibrium [10,18].

Whereas no significant differences were observed between groups regarding body posture characteristics, the asthma group displayed slight variations in body posture compared to the healthy control group, particularly evident in kyphotic and lordotic angles. Similar findings regarding body posture variables in asthma patients have been reported by other researchers. Adults with chronic asthma commonly exhibit postural abnormalities, attributed to a hyperinflated thorax causing compensations in the thoracic spine, pelvic girdle, and scapular belt due to alterations and shortening of the diaphragm. Thoracic hyperinflation may contribute to an increase in thoracic kyphosis, given the direct attachment of the diaphragm to the endothoracic fascia [9,31,32].

Our study revealed an enhancement in thoracic kyphosis, potentially attributed to the attachment of the diaphragm to other thoracic muscles, leading to pelvic anteversion and lumbar hyperlordosis, as observed in our asthma patient sample. These findings are consistent with those reported by Almeida et al., who identified unique postural issues in asthma patients. Garreau de Loubresse and Wolff noted a close relationship between thoracic kyphotic angle and lung function [9,33,34]. Recent studies have highlighted the occurrence of hyperinflation even in medicated and asymptomatic patients with mild to moderate asthma, suggesting that disease stabilization through symptom management does not necessarily mitigate hyperinflation, which may contribute to body postural alterations [34].

Our study identified a mild to moderate positive correlation between pulmonary function parameters (FEV1%, FVC%, PEFR) and postural balance metrics (overall stability index, anterior-posterior stability index, and medial-lateral stability index) during double leg eye closure. Additionally, a weak to moderate negative correlation was observed between pulmonary function (FEV1/FVC%) and postural balance. We believe the reduced contribution of the thoracic region to postural sway may be associated with increased trunk stiffness and decreased flexibility of the thoracic spine, rib cage, and sternum. Consequently, individuals with asthma rely on lumbar, hip, and ankle mobility to mitigate the shift in the center of gravity [10].

The study’s findings focused on young adults with childhood-onset asthma aged 20 to 25, limiting generalizability to other age groups. Peripheral muscle strength and medication types commonly used in asthma were not assessed, potentially confounding the results. As a cross-sectional study, it only establishes associations rather than cause-effect relationships. The clinical significance of this study lies in its demonstration that young adults with childhood-onset asthma, even in mild to moderate cases, exhibit impaired pulmonary function and reduced respiratory muscle strength compared to healthy peers. However, the study found no significant differences in postural balance or body posture between the groups. These findings suggest that clinicians should focus on monitoring and managing respiratory function in young adults with asthma, while routine postural and balance assessments may not be necessary for all patients with mild to moderate asthma.

The study has some limitations. First, because most individuals in our study had mild to moderate asthma, our findings cannot be applied routinely. Second, we did not test all adult age groups for asthma, instead focusing on childhood asthma aged 18–25. Third, we did not assess peripheral muscle strength, or the type of medication typically administered to individuals with asthma, which could have been a confounding factor. Furthermore, because this is a cross-sectional study, it simply reveals associations and does not demonstrate a cause-effect relationship. Future research could explore postural balance, body posture, and peripheral muscular strength in older adults with moderate to severe asthma to provide a more comprehensive understanding of the topic.

## 5. Conclusions

In young adults with childhood-onset mild to moderate asthma, we found impacts of asthma on pulmonary function and respiratory muscle strength but not in postural balance and body posture compared to healthy controls. While we identified a weak to moderate correlation between pulmonary function and specific postural balance variables, there was no correlation between respiratory function and overall postural balance or body posture.

## 6. Clinical implications

The practical implications of this study are significant for clinical practice and asthma management. Regular monitoring of pulmonary function in young adults with childhood-onset asthma is crucial, as the study found notable differences in respiratory function compared to healthy peers. Incorporating respiratory muscle training into treatment plans could enhance respiratory strength, addressing the observed deficits in muscle function. Additionally, while no significant differences in postural balance were noted, assessing balance in asthma patients may still be beneficial, particularly for those with more severe symptoms. Educating patients about maintaining proper posture can help mitigate potential long-term complications associated with asthma. Overall, the findings underscore the importance of a comprehensive approach to asthma management that considers both respiratory health and physical function, ultimately aiming to improve the quality of life for young adults living with this condition.

## Supporting information

S1 Table Postural analysis parameters and their definitions. (DOCX)

S2 Table Correlation between pulmonary function, postural balance, and body posture. (DOCX)
