# Supplementary material for: Pulmonary function, body posture and balance in young adults with asthma: A cross-sectional study
Source: PLoS One. 2025 Mar 3;20(3):e0316663. doi: 10.1371/journal.pone.0316663 (PMC11875369; doi:10.1371/journal.pone.0316663)
Supplement: S1 Table — (DOCX) [file pone.0316663.s001.docx]

**Supplementary Table 1: Postural analysis parameters and their definitions.**

| **Variable** | **Definition** |
| --- | --- |
| **Localization and distance** | |
| - Trunk length, mm | - The distance from VP to DM |
| - Dimple distance, mm | - The distance from DL to DR |
| **Trunk and pelvis imbalance** | |
| - Sagittal imbalance (trunk inclination), degree - Coronal imbalance (trunk imbalance), mm - Pelvic obliquity (pelvic tilt), mm - Pelvic torsion, degree | - The angle formed by an external plumb line and the line that connects VP and DM.   Laterally estimated distance between VP and DM   - Difference in height between DL and DR - Surface normal torsions of DL and DR |
| **Spinal curve measurements** | |
| - Kyphotic angle (max), degree | - Angle formed between the surface tangents from ICT and ITL |
| - Lordotic angle (max), degree | - Angle formed between the surface tangents from ITL and ILS |
| **Spinal deviation** | |
| - Vertebral rotation (+max) (surface rotation) - Vertebral rotation (rms) (surface rotation) - Vertebral rotation (amplitude) (surface rotation) | - The highest value of the horizontal components of the surface normal on the right-hand symmetry - RMS of the horizontal components of the surface normal on the symmetry line - Maximum spinal torsion estimated from maximum rotations to the right and left |

DM: the center point between sacral dimple on the left and sacral dimple on the right; DL: sacral dimple left; DR: sacral dimple right; ICT: cervicothoracic transition point; ILS: lumbosacral transition point; ITL: thoracolumbar transition point; VP: vertebral prominence
