# Supplementary material for: Pulmonary function, body posture and balance in young adults with asthma: A cross-sectional study
Source: PLoS One. 2025 Mar 3;20(3):e0316663. doi: 10.1371/journal.pone.0316663 (PMC11875369; doi:10.1371/journal.pone.0316663)
Supplement: S2 Table — (DOCX) [file pone.0316663.s002.docx]

**Table 2S: Correlation between pulmonary function, postural balance, and body posture**.

| **Variables** | **Correlation (r)** | **Lower. Confidence interval (CI)** | **Upper. confidence interval (CI)** | **p-value** |
| --- | --- | --- | --- | --- |
| **Pulmonary function vs postural balance** | | | | |
| FEV_1_ (%) vs 0SI- EC | 0.43 | 0.27 | 0.56 | <0.001 |
| FVC (%) vs 0SI- EC | 0.43 | 0.28 | 0.57 | <0.001 |
| FEV_1_/FVC % vs 0SI- EC | -0.42 | -0.56 | -0.27 | <0.001 |
| FEV_1_ (%) vs APSI-EC | 0.40 | 0.24 | 0.54 | <0.001 |
| FVC (%) vs APSI-EC | 0.41 | 0.25 | 0.55 | <0.001 |
| FEV1/FVC % vs APSI-EC | -0.38 | -0.52 | -0.22 | <0.001 |
| FEV_1_ (%) vs MLSI-EC | 0.38 | 0.22 | 0.52 | <0.001 |
| FVC (%) vs MLSI-EC | 0.39 | 0.22 | 053 | <0.001 |
| FEV_1_/FVC % vs MLSI-EC | -0.42 | -0.55 | -0.26 | <0.001 |
| **PEFR vs MLSI-EC** | 0.19 | 0.01 | 0.35 | 0.03 |
| **Pulmonary function vs body posture** | | | | |
| FEV_1_%vs Trunk length | 0.60 | 0.47 | 0.70 | <0.001 |
| FVC % vs Trunk length | 0.60 | 0.47 | 0.70 | <0.001 |
| FEV1/FVC % vs Trunk length | -0.58 | -0.69 | -0.45 | <0.001 |
| PEFR vs Trunk length | 0.18 | 0.01 | 0.35 | 0.04 |
| FEV_1_ vs lordotic angle | -0.22 | -0.38 | -0.04 | 0.01 |
| FVC vs lordotic angle | -0.21 | -0.38 | -0.04 | 0.01 |
| FEV_1_/FVC % vs lordotic angle | 0.18 | 0.01 | 0.35 | 0.04 |

0SI- EC: 0verall stability index -EYES CLOSED, APSI-EC: Anterior-posterior stability index -EYES CLOSED, MLSI-EC: Medial-lateral stability index - EYES CLOSED, FVC =Forced vital capacity, FEV_1_=Forced expiratory volume in the first second, PEFR=Peak expiratory flow rate
