# Supplementary material for: Pulmonary function, body posture and balance in young adults with asthma: A cross-sectional study
Source: PLoS One. 2025 Mar 3;20(3):e0316663. doi: 10.1371/journal.pone.0316663 (PMC11875369; doi:10.1371/journal.pone.0316663)
Supplement: Attachment — Submitted filename: Reviewer comment & Author response.docx [file pone.0316663.s003.docx]

| **Reviewer comment--1** | **Author response:** | **Page Number** |
| --- | --- | --- |
| In the abstract and full text, you should only say cross-sectional study (correlational study is not a familiar and acceptable term). | Thank you for this suggestion. We agree and have revise the terminology throughout the manuscript to consistently use "cross-sectional study" instead of "correlational cross-sectional study | 2 |
| On page 3, the sentence "A study exploring the relationship between pulmonary function and postural balance revealed a limited understanding of this subject" with reference number 10 should be placed at the last paragraph of introduction where you mention current evidence limitations in asthma | We appreciate this recommendation. We have moved this sentence to the last paragraph of the introduction to better highlight the current limitations in the literature regarding asthma. | 4 |
| Before the aim of the study, it should be clearly stated what the study will contribute to the literature. Was it only the objective measurements related to balance or posture that were missing in young asthma? | Thank you for pointing this out. We have added a clear statement about the study's contribution to the literature, emphasizing that our study provides objective measurements of both balance and posture in young adults with childhood-onset asthma, which were previously lacking in the literature. | 4 |
| You can say "determine" rather than "describe" in the aim. Also, you can say "matched groups in terms of physical measures." You repeat age, gender, body weight and height two times. | We agree with these suggestions. We have revise the aim to use "determine" instead of "describe" and will simplify the description of matched groups to avoid repetition. | 4 |
| On page 5, the sentence "Statistical analysis was performed at the Augusto Motta University Center, Brazil" should be removed. | We have remove this sentence as suggested. | 5 |
| You should add "years" after ages in the full paper. | We have added "years" after all age mentions throughout the manuscript for clarity. | 5 |
| I think you should use either "healthy" or "control," not both of them. | We appreciate this observation. We have consistently use "control group" throughout the manuscript to maintain clarity and consistency. |  |
| On page 5, you only say age and gender matched, opposite to the introduction part. You should check. | Thank you for catching this inconsistency. We have revised the manuscript to ensure consistent description of matching criteria (age, gender, weight, and height) throughout. | 5 |
| In the assessment part, you should only give the order of assessment and total duration of assessments. Details about measurement were placed in the detailed description of all outcomes separately. | We have restructured this section as suggested, providing only the order and total duration of assessments, and move specific measurement details to their respective section | 6 |
| On page 7, you should mention you interpreted respiratory muscle results as percentage of expected values. | We appreciate the reviewer's comment. However, we would like to clarify that in our study, we did not interpret respiratory muscle strength results as percentages of predicted values. Instead, as stated in the manuscript, we used a different criterion for data analysis. Specifically, we considered a measurement valid if the highest value did not exceed the second highest value by 10%. We will ensure this is clearly stated in the manuscript to avoid any confusion. |  |
| On page 9, sample size section should be embedded into statistical analysis section. | We have moved the sample size calculation details into the statistical analysis section as suggested | 10 |
| On page 10, for Pearson correlation analysis, you should say "to investigate correlation of dynamic balance and body posture with respiratory function measures." | We have revised this description as suggested to more accurately reflect the correlation analysis performed. | 10 |
| You should add flow chart in the results section. | We have created and include a flow chart in the results section to illustrate participant recruitment and selection. | 11 |
| After full corrections, full paper should be checked with a grammar edit program. | We have conduct a thorough grammar check using a professional editing tool after implementing all revisions |  |
| The clinical significance and contribution of these results should be stated in the last part of the first paragraph of the discussion | We have add a statement about the clinical significance and contribution of our findings at the end of the first discussion paragraph. | 15 |
| The sentence "These findings contradict clinical evidence suggesting alterations in postural balance and body posture among young adults with mild to moderate asthma" should be embedded in the discussion with reasons. | We have incorporate this sentence into the discussion, providing potential explanations for the contradictory findings. | 14,15 |
| On second paragraph, the difference in expiratory muscle strength should be discussed in detail. (You only mention MIP values) | We have expand the discussion to include a more detailed analysis of both inspiratory (MIP) and expiratory (MEP) muscle strength differences between groups. | 13 |
| I think other limitations of the study that not generalisability for severe asthma you should add. | We have add a statement in the limitations of the study section acknowledging that our findings may not be generalizable to individuals with severe asthma. | 15 |
| At the end of conclusion, you should give advice for pulmonary rehabilitation clinics and academicians for future studies. You only repeat your results. | Thank you for your insightful comment. We appreciate the suggestion to include recommendations for pulmonary rehabilitation clinics and future research directions. However, we would like to clarify that our study was primarily an assessment study focusing on postural balance and body posture in young adults with asthma. As our results did not show significant differences in these parameters between the asthma and control groups, we did not include specific recommendations for pulmonary rehabilitation. We Add a paragraph discussing the potential clinical applications and implications of the findings in the discussion section | 16 |
| Figure 2 is not understandable, it should either be removed or details should be given. | We have review Figure 2 and provide more detailed explanation |  |
| Significant values in the tables should be written in bold or expressed with a symbol. | We have revise all tables to highlight significant values by bolding them to indicate statistical significance. |  |
| **Reviewer comment--2** | **Author response:** | **Page number** |
| Abstract  - Please better state the results obtained in the abstract. | We have Revised the abstract to include specific numerical results, p-values, for significant differences between groups, particularly for pulmonary function variables (FEV1, FVC, PEFR) and respiratory muscle strength (MEP). | 2 |
| Introduction  - The introduction needs to be supported by more current references from last years. | Incorporate recent studies (published within the last 3-5 years) on asthma, postural balance, and body posture in the introduction to provide up-to-date context. |  |
| “A study exploring the relationship between pulmonary function and postural balance revealed a limited understanding of this subject” - Please clarify this issue. | Specifically, previous studies have often lacked comprehensive methodologies that adequately assess both pulmonary function and postural balance in a systematic manner. Many existing studies either focus solely on one aspect (e.g., pulmonary function) or do not explore the interplay between respiratory mechanics and balance control.  Additionally, there is a scarcity of research that examines how variations in pulmonary function impact specific balance parameters, particularly in populations with childhood-onset asthma. While some studies suggest that asthma can lead to musculoskeletal dysfunction and postural abnormalities, they do not sufficiently elucidate the mechanisms linking these factors to balance impairments.  Our research aims to fill these gaps by systematically investigating the relationships between pulmonary function parameters (such as FEV1, FVC, and respiratory muscle strength) and various aspects of postural balance in young adults with childhood-onset asthma. By employing rigorous assessment techniques and a well-defined participant group, we hope to provide clearer insights into how respiratory health influences postural stability, thereby contributing to a more nuanced understanding of this critical area of study. |  |
| Authors should emphasize the novelty of their work. | the unique aspects of this study, such as the combination of pulmonary function, body posture, and dynamic balance assessments in young adults with childhood-onset asthma. |  |
| The authors should be better specified, at the end of the introduction section, the rationale of the study and the aim of the study. | Create a separate paragraph at the end of the introduction that clearly states the rationale and aims of the study | 8 |
| State the hypothesis of the study at the end of the introduction. | Add a clear hypothesis predicting the expected differences between asthma and control groups and the relationships between pulmonary function, body posture, and dynamic balance. | 8 |
| Material and Methods: Where and how were participants recruited? | Provide more details on the specific recruitment process used at the College of Health Sciences, University of Sharjah.aged 18 to 25 years with mild to moderate asthma since childhood, referred by a pulmonologist or physician, medically stable, and without exacerbations within the previous 6 weeks. |  |
| Provide the measurement reliability of the MIR-Spiro Lab Spirometer and the other used tools. | Include information on the reliability and validity of all measurement tools, citing relevant studies | 7 |
| Please better organize the description of the measurements conducted, for example, by including them in a shared subsection such as 'Outcomes' | Reorganize the methods section with clear subsections for each measurement type to improve readability. We have put heading Description of Outcome Measures | 6 |
| The duration of the balance measurement was set at 20 seconds. Why exactly this duration? | The 20-second duration for balance measurement is a standard procedure for the Biodex Balance System. This duration has been established as an optimal time frame to assess postural stability while minimizing fatigue effects. Previous studies using the Biodex Balance System have consistently employed this 20-second testing protocol, demonstrating its reliability and validity in assessing balance performance. By adhering to this standardized duration, our study ensures comparability with existing literature and maintains consistency in balance assessment methodology. |  |
| Please add manufacturer and country next to the devices (The Biodex Balance System, DIERS Formetric 4D® sensors). | Add manufacturer and country information for all devices mentioned in the methods section. | 7,8 |
| Was the distribution of the assessed variables examined? | Include information on normality tests or other distribution assessments in the statistical analysis section. | 10 |
| "These findings contradict clinical evidence suggesting alterations in postural balance and body posture among young adults with mild to moderate asthma." - What could be the cause of this discrepancy in the results? | The discrepancy between our findings and previous studies may be due to several factors: Asthma Severity: Our study focused on young adults with mild to moderate asthma, while other studies may have included participants with more severe asthma, which could lead to greater postural and balance impairments. Measurement Techniques: We utilized advanced tools like the Biodex Balance System for balance assessment, which may provide more accurate measurements compared to methods used in earlier studies. Sample Characteristics: Differences in age, duration of asthma, and overall health status of participants in our study compared to others could influence the results. Management of Asthma: Participants in our study were medically stable and receiving appropriate treatment, which may mitigate some of the postural and balance issues observed in other research. |  |
| "Hyperinflation in asthma may lead to the flattening of the diaphragm, resulting in mechanical disadvantages and reduced function of the inspiratory muscles." – Please add references. | Add relevant references supporting this statement about hyperinflation and diaphragm function in asthma | 15 |
| The results should not be overinterpreted. The differences between the groups were not statistically significant, and the effect size was trivial | Revise the discussion to accurately reflect the statistical findings, avoiding overinterpretation of non-significant results. | 12-15 |
| Another potential explanation for decreased postural balance in asthma patients is their heightened reliance on visual input for balance control on a mobile platform." - Please explain this hypothesis. | We have added this statement in the discussion “Due to potential deficits in motor systems, they may use visual information more heavily to compensate for instability, which could make balance more challenging in environments where visual input is limited or altered." | 14 |
| Recent studies have highlighted the occurrence of hyperinflation even in medicated and asymptomatic patients with mild to moderate asthma, suggesting that disease stabilization through symptom management does not necessarily mitigate hyperinflation, which may contribute to body postural alterations." - please add references. | Add citations to relevant recent studies supporting this statement about hyperinflation in medicated and asymptomatic asthma patients. |  |
| Similar to the introduction, the innovation of this work should be emphasized. What gap in knowledge do the study fill? | In the conclusion we clearly state how this study contributes new knowledge to the field, addressing previously unanswered questions about the relationships between pulmonary function, body posture, and dynamic balance in young adults with childhood-onset asthma. | 16 |
| Please state the practical implications of your study. | Add a paragraph discussing the potential clinical applications and implications of the findings in the discussion section | 16 |
