# Supplementary material for: Pulmonary function, body posture and balance in young adults with asthma: A cross-sectional study
Source: PLoS One. 2025 Mar 3;20(3):e0316663. doi: 10.1371/journal.pone.0316663 (PMC11875369; doi:10.1371/journal.pone.0316663)
Supplement: Peer review history

# Author comment: Author response to Decision Letter 0

**DOI:** 10.1371/journal.pone.0316663.r001

17 Aug 2024

# Peer review: Decision Letter 0

**DOI:** 10.1371/journal.pone.0316663.r002

**Authors:** Barry Kweh

3 Dec 2024

Dear Dr. Alaparthi,

Thank you for submitting your manuscript to PLOS ONE. After careful consideration, we feel that it has merit but does not fully meet PLOS ONE’s publication criteria as it currently stands. Therefore, we invite you to submit a revised version of the manuscript that addresses the points raised during the review process.

Please submit your revised manuscript by Jan 17 2025 11:59PM. If you will need more time than this to complete your revisions, please reply to this message or contact the journal office at plosone@plos.org . When you're ready to submit your revision, log on to https://www.editorialmanager.com/pone/ and select the 'Submissions Needing Revision' folder to locate your manuscript file.

- A rebuttal letter that responds to each point raised by the academic editor and reviewer(s). You should upload this letter as a separate file labeled 'Response to Reviewers'.
- A marked-up copy of your manuscript that highlights changes made to the original version. You should upload this as a separate file labeled 'Revised Manuscript with Track Changes'.
- An unmarked version of your revised paper without tracked changes. You should upload this as a separate file labeled 'Manuscript'.

If you would like to make changes to your financial disclosure, please include your updated statement in your cover letter. Guidelines for resubmitting your figure files are available below the reviewer comments at the end of this letter.

If applicable, we recommend that you deposit your laboratory protocols in protocols.io to enhance the reproducibility of your results. Protocols.io assigns your protocol its own identifier (DOI) so that it can be cited independently in the future. For instructions see: https://journals.plos.org/plosone/s/submission-guidelines#loc-laboratory-protocols . Additionally, PLOS ONE offers an option for publishing peer-reviewed Lab Protocol articles, which describe protocols hosted on protocols.io. Read more information on sharing protocols at https://plos.org/protocols?utm_medium=editorial-email&utm_source=authorletters&utm_campaign=protocols .

We look forward to receiving your revised manuscript.

Kind regards,

Barry Kweh

Academic Editor

PLOS ONE

Journal Requirements:

When submitting your revision, we need you to address these additional requirements.

1. Please ensure that your manuscript meets PLOS ONE's style requirements, including those for file naming. The PLOS ONE style templates can be found at https://journals.plos.org/plosone/s/file?id=wjVg/PLOSOne_formatting_sample_main_body.pdf and https://journals.plos.org/plosone/s/file?id=ba62/PLOSOne_formatting_sample_title_authors_affiliations.pdf

2. Please note that PLOS ONE has specific guidelines on code sharing for submissions in which author-generated code underpins the findings in the manuscript. In these cases, we expect all author-generated code to be made available without restrictions upon publication of the work. Please review our guidelines at https://journals.plos.org/plosone/s/materials-and-software-sharing#loc-sharing-code and ensure that your code is shared in a way that follows best practice and facilitates reproducibility and reuse.

3. Please note that funding information should not appear in any section or other areas of your manuscript. We will only publish funding information present in the Funding Statement section of the online submission form. Please remove any funding-related text from the manuscript.

4. Thank you for stating the following financial disclosure: “This study was supported by the Fundação Carlos Chagas Filho de Apoio à Pesquisa do Estado do Rio de Janeiro (FAPERJ, No. E-26/211.104/2021) and Coordenação de Aperfeiçoamento de Pessoal (CAPES, Finance Code 001; No. 88881.708719/2022-01, and No. 88887.708718/2022-00).”

Please state what role the funders took in the study. If the funders had no role, please state: "The funders had no role in study design, data collection and analysis, decision to publish, or preparation of the manuscript." If this statement is not correct you must amend it as needed. Please include this amended Role of Funder statement in your cover letter; we will change the online submission form on your behalf.

5. We note that your Data Availability Statement is currently as follows: “All relevant data are within the manuscript and in Supporting Information files.”

Please confirm at this time whether or not your submission contains all raw data required to replicate the results of your study. Authors must share the “minimal data set” for their submission. PLOS defines the minimal data set to consist of the data required to replicate all study findings reported in the article, as well as related metadata and methods (https://journals.plos.org/plosone/s/data-availability#loc-minimal-data-set-definition). For example, authors should submit the following data: - The values behind the means, standard deviations and other measures reported; - The values used to build graphs; - The points extracted from images for analysis. Authors do not need to submit their entire data set if only a portion of the data was used in the reported study. If your submission does not contain these data, please either upload them as Supporting Information files or deposit them to a stable, public repository and provide us with the relevant URLs, DOIs, or accession numbers. For a list of recommended repositories, please see https://journals.plos.org/plosone/s/recommended-repositories. If there are ethical or legal restrictions on sharing a de-identified data set, please explain them in detail (e.g., data contain potentially sensitive information, data are owned by a third-party organization, etc.) and who has imposed them (e.g., an ethics committee). Please also provide contact information for a data access committee, ethics committee, or other institutional body to which data requests may be sent. If data are owned by a third party, please indicate how others may request data access.

6. PLOS requires an ORCID iD for the corresponding author in Editorial Manager on papers submitted after December 6th, 2016. Please ensure that you have an ORCID iD and that it is validated in Editorial Manager. To do this, go to ‘Update my Information’ (in the upper left-hand corner of the main menu), and click on the Fetch/Validate link next to the ORCID field. This will take you to the ORCID site and allow you to create a new iD or authenticate a pre-existing iD in Editorial Manager.

Additional Editor Comments:

The authors have written a well written manuscript but, as noted by reviewers, need to clarify their methodology including method of recruitment. Their discussion should also incorporate why they believe their findings are similar and different from the literature, and why they believe there are some contradictions with clinical evidence.

[Note: HTML markup is below. Please do not edit.]

Reviewers' comments:

Reviewer's Responses to Questions

**Comments to the Author**

1. Is the manuscript technically sound, and do the data support the conclusions?

Reviewer #1: Yes

Reviewer #2: Partly

**********

2. Has the statistical analysis been performed appropriately and rigorously?

Reviewer #1: Yes

Reviewer #2: Yes

**********

3. Have the authors made all data underlying the findings in their manuscript fully available?

The PLOS Data policy

Reviewer #1: No

Reviewer #2: Yes

**********

4. Is the manuscript presented in an intelligible fashion and written in standard English?

Reviewer #1: Yes

Reviewer #2: Yes

**********

Reviewer #1: Dear authors

Thank you very much for your valuable contribution

I have some minor concerns:

1. In the asbtract and full text, you only should say cross sectional study (correlational study is not familiar and acceptable term)

2. On page 3, sentence (A study exploring the

relationship between pulmonary function and postural balance revealed a limited understanding of this subject) with reference number 10 should be placed at the last paragraph of introduction where you mention current evidence limitations in asthma

3. Before the aim of the study, it should be clearly stated what the study will contribute to the literature. Was it only the objective measurements related to balance or posture that were missing in young asthma?

4. You can say determine rather than describe in the aim. Also you can say matched groups in terms of physical measures. You repeat age, gender, body weight and height for two times.

5. Onpage 5, the sentence should be removed (Statistical analysis was performed at the Augusto Motta University Center, Brazil.)

6. You should add years after ages in the full paper.

7. I think you should use healthy or control not both of them

8. On page 5 you only say age and gender matched opposite to introduction part. You should check.

9. In the assesment part, you should only give the order of assesment and total duration of assesments. Details about measurement were placed in the detailed description of all outcomes seperately

10. On page 7, you should mention you interpreted respiratory muscle results as percentage of expected values

11. On page 9, sample size section should be embedded into statistical analysis section

12. On page 10, for pearson correlation analysis you should say to investigate correlation of dynamic balance and body posture with respiratory function measures.

13. You should add flow chart in the results section.

14. After full corrections, full paper should be checked with a grammer edit programme.

15. The clinical significance and contribution of these results should be stated in the last part of the first paragraph of the discussion.

16. 'These findings contradict clinical evidence suggesting alterations in postural balance and body posture among young adults with mild to moderate asthma' sentence should be embedded in to discusssion with reasons

17. On second paragraph, the difference in expiratory muscle strength should be discusssed in detail. (you only mention MİP values)

18. I think other limitations of the study that not generalisability for severe asthma you should add.

19. At the end of conclusion, you should give advice for pulmonary rehabilitation clinics and academicians for future studies. You only repeat your results.

20. Figure 2 is not understandable, it should either be removed or details should be given.

21. Significant values in the tables should be written in bold or expressed with a symbol.

sincerely

18.

Reviewer #2: Numbering the lines in the manuscript would be helpful.

Abstract

- Please better state the results obtained in the abstract.

Introduction

- The introduction needs to be supported by more current references from last years.

- “A study exploring the relationship between pulmonary function and postural balance revealed a limited understanding of this subject” - Please clarify this issue.

- In my opinion Authors should emphasize the novelty of their work.

- The authors should be better specified, at the end of the introduction section, the rationale of the study and the aim of the study.

- State the hypothesis of the study at the end of the introduction.

Material and Methods

- Where and how were participants recruited?

- Provide the measurement reliability of the MIR-Spiro Lab Spirometer and the other used tools.

- Please better organize the description of the measurements conducted, for example, by including them in a shared subsection such as 'Outcomes'.

- The duration of the balance measurement was set at 20 seconds. Why exactly this duration?

- Please add manufacturer and country next to the devices (The Biodex Balance System, DIERS Formetric 4D® sensors).

- Was the distribution of the assessed variables examined?

Discussion

- “These findings contradict clinical evidence suggesting alterations in postural balance and body posture among young adults with mild to moderate asthma.” - What could be the cause of this discrepancy in the results?

- “Hyperinflation in asthma may lead to the flattening of the diaphragm, resulting in mechanical disadvantages and reduced function of the inspiratory muscles.” – Please add references.

- “The asthma group exhibited lower postural balance compared to the healthy control group, particularly evident during the double leg support, and eye closed condition, as indicated by lower scores in the overall stability index, anterior-posterior stability index, and medial-lateral stability index. This impairment in postural balance among asthma patients may be attributed to the degree of hyperinflation, which could lead to increased activation of trunk muscles and subsequent rigidity. This heightened muscular activation may diminish the significance of trunk motion and force moments in balance control” - The results should not be overinterpreted. The differences between the groups were not statistically significant, and the effect size was trivial.

- “Another potential explanation for decreased postural balance in asthma patients is their heightened reliance on visual input for balance control on a mobile platform.” - Please explain this hypothesis.

- “Recent studies have highlighted the occurrence of hyperinflation even in medicated and asymptomatic patients with mild to moderate asthma, suggesting that disease stabilization through symptom management does not necessarily mitigate hyperinflation, which may contribute to body postural alterations.” - please add references.

- Similar to the introduction, the innovation of this work should be emphasized. What gap in knowledge do the study fill?

- Please state the practical implications of your study.

**********

what does this mean? ). If published, this will include your full peer review and any attached files.

If you choose “no”, your identity will remain anonymous but your review may still be made public.

**Do you want your identity to be public for this peer review?** For information about this choice, including consent withdrawal, please see our Privacy Policy

Reviewer #1: **Yes: ** Ebru Calik Kutukcu

Reviewer #2: No

**********

[NOTE: If reviewer comments were submitted as an attachment file, they will be attached to this email and accessible via the submission site. Please log into your account, locate the manuscript record, and check for the action link "View Attachments". If this link does not appear, there are no attachment files.]

While revising your submission, please upload your figure files to the Preflight Analysis and Conversion Engine (PACE) digital diagnostic tool, https://pacev2.apexcovantage.com/ . PACE helps ensure that figures meet PLOS requirements. To use PACE, you must first register as a user. Registration is free. Then, login and navigate to the UPLOAD tab, where you will find detailed instructions on how to use the tool. If you encounter any issues or have any questions when using PACE, please email PLOS at figures@plos.org . Please note that Supporting Information files do not need this step.

# Author comment: Author response to Decision Letter 0

**DOI:** 10.1371/journal.pone.0316663.r003

13 Dec 2024

All the comments were addressed and incorporated throughout the manuscript and also attached a detailed response to reviewer file.

Attachment Submitted filename: Reviewer comment & Author response.docx

# Editor report: Decision Letter 1

**DOI:** 10.1371/journal.pone.0316663.r004

**Authors:** Barry Kweh

16 Dec 2024

Pulmonary function, body posture and balance in young adults with asthma: A cross-sectional study

PONE-D-24-33304R1

Dear Dr. Alaparthi,

We’re pleased to inform you that your manuscript has been judged scientifically suitable for publication and will be formally accepted for publication once it meets all outstanding technical requirements.

Within one week, you’ll receive an e-mail detailing the required amendments. When these have been addressed, you’ll receive a formal acceptance letter and your manuscript will be scheduled for publication.

An invoice will be generated when your article is formally accepted. Please note, if your institution has a publishing partnership with PLOS and your article meets the relevant criteria, all or part of your publication costs will be covered. Please make sure your user information is up-to-date by logging into Editorial Manager at Editorial Manager®  and clicking the ‘Update My Information' link at the top of the page. If you have any questions relating to publication charges, please contact our Author Billing department directly at authorbilling@plos.org.

If your institution or institutions have a press office, please notify them about your upcoming paper to help maximize its impact. If they’ll be preparing press materials, please inform our press team as soon as possible -- no later than 48 hours after receiving the formal acceptance. Your manuscript will remain under strict press embargo until 2 pm Eastern Time on the date of publication. For more information, please contact onepress@plos.org.

Kind regards,

Barry Kweh

Academic Editor

PLOS ONE

Additional Editor Comments (optional):

The authors have significantly improved the structure of their manuscripts. The primary objective of investigating asthma's effect on young adults in various domains is clearly stated. Several methodological queries raised by the reviewers have been addressed, the discussion is more expansive and limitations are now more clearly acknowledged.

Reviewers' comments:

# Editor report: Acceptance letter

**DOI:** 10.1371/journal.pone.0316663.r005

**Authors:** Barry Kweh

PONE-D-24-33304R1

PLOS ONE

Dear Dr. Alaparthi,

I'm pleased to inform you that your manuscript has been deemed suitable for publication in PLOS ONE. Congratulations! Your manuscript is now being handed over to our production team.

At this stage, our production department will prepare your paper for publication. This includes ensuring the following:

* All references, tables, and figures are properly cited

* All relevant supporting information is included in the manuscript submission,

* There are no issues that prevent the paper from being properly typeset

If revisions are needed, the production department will contact you directly to resolve them. If no revisions are needed, you will receive an email when the publication date has been set. At this time, we do not offer pre-publication proofs to authors during production of the accepted work. Please keep in mind that we are working through a large volume of accepted articles, so please give us a few weeks to review your paper and let you know the next and final steps.

Lastly, if your institution or institutions have a press office, please let them know about your upcoming paper now to help maximize its impact. If they'll be preparing press materials, please inform our press team within the next 48 hours. Your manuscript will remain under strict press embargo until 2 pm Eastern Time on the date of publication. For more information, please contact onepress@plos.org.

If we can help with anything else, please email us at customercare@plos.org.

Thank you for submitting your work to PLOS ONE and supporting open access.

Kind regards,

PLOS ONE Editorial Office Staff

on behalf of

Dr. Barry Kweh

Academic Editor

PLOS ONE
